# Exploring the role of Dynamic Presencing in a group coaching training context for fostering transformative leadership development in disruptive times

**DOI:** 10.3389/fpsyg.2024.1352828

**Published:** 2024-05-15

**Authors:** Cecile Gerwel Proches, Preeya Daya, Jessica Strayer, Cristy Leask, Ntokozo Mngadi, Christa de Lange, Olen Gunnlaugson

**Affiliations:** ^1^Graduate School of Business and Leadership, University of KwaZulu-Natal, Durban, South Africa; ^2^Graduate School of Business, University of Cape Town, Cape Town, South Africa; ^3^Jessica J. Strayer Consulting, LLC, Woodbury, MN, United States; ^4^HCA Healthcare, London, United Kingdom; ^5^FSA – Université Laval, Québec City, QC, Canada

**Keywords:** Dynamic Presencing, leadership, VUCA, transformative leadership, inner leadership, group coaching

## Abstract

With the growing array of challenges humanity has been experiencing since the global pandemic, knowledge workers at all levels of organizations are experiencing a noted increase in the volatility, uncertainty, complexity, and ambiguity (VUCA) conditions of their work and lives. This has brought about significant changes in ways of working and associated ways of being that have become more fragmented and virtual. Disruptive change continues to unfold on many levels of society, from the organizational to the individual level, with multiple and often unintended consequences. This article outlines how the body of work of Dynamic Presencing in a group coaching context facilitates responsive forms of personal development as well as a deeper transformation of one’s leadership identity in the face of such change, which in turn serves as an approach that can be useful in navigating VUCA conditions that are becoming increasingly prevalent. The transformative process of Dynamic Presencing develops core inner leadership capabilities with a noted increase in the quality of internal and interpersonal forms of self-,presence-, and presencing-awareness. Overall, Dynamic Presencing serves as a critical enabler of presencing mastery, which instills deeper confidence and resourcefulness in working with the VUCA conditions of our global world by deepening our presencing process and refining our methods for inner leadership development in turbulent times.

## 1 Introduction

The COVID-19 pandemic recently brought about significant disruption across the globe, amplifying VUCA conditions (i.e., increased volatility, uncertainty, complexity, and ambiguity) in most organizational and societal contexts. These disruptive challenges resulted in increased uncertainty, isolation, and anxiety, impacting existential concerns regarding health and wellbeing, including physical illness and mental illness, such as loneliness and depression. This global event challenged traditional transactional forms of leadership that are power/expertise-based authoritarian styles applied in predictable and more controllable periods, opening pathways in more progressive contexts for experimenting with post-conventional leadership styles, including servant leadership (Keith, 2023), responsible leadership (Haider et al., 2022), and authentic leadership (Sarkar, 2016), among others, that we broadly characterize as transformative approaches.

Collectively these leadership approaches aspired to be visionary, cultivating honesty, integrity, trustworthiness, being in-service of others, role-modelling, pioneering, appreciating others, empowering, genuine and self-aware (Russell and Stone, 2002; Sarkar, 2016). In addition to this overall development, the increasing need for leaders to be well-grounded and resourced in the present moment (Gunnlaugson, 2020a) has necessitated the cultivation of transformative leadership approaches that draw from introspection, personal reflection, and a more developed awareness of the inner place and identity from which the leader leads. To sum up, one could conclude that, in a post-pandemic world, post-conventional leadership approaches are needed to effectively engage the complex leadership challenges of this current historical moment. Overall, the global pandemic demonstrated a new imperative for how individuals at various levels of leadership need to rise to the occasion to be adaptable, resilient, and embrace the full force of change while retaining an openness and humility to learning new transformational paths of self-leadership that have little precedent. In demanding responsive and transformative leadership at multiple levels, the global pandemic presented an opportunity for people from all levels of the organization to explore emerging capabilities to be proactive in leading and navigating change (Korejan and Shahbazi, 2016).

### 1.1 Transforming leader identity

In this article, we view transformational leadership processes as critical for leadership development. Particularly those transformational leadership approaches that focus on cultivating the inner consciousness dimensions of a leader’s identity by developing their self-awareness and presence development (i.e., such elements as internal forms of self-awareness, confidence, and resourcefulness), and, in so doing, helping leaders be resourced and grounded to learn to lead from an inner place of wisdom, stillness, and an authentic meeting of one’s self through the group coaching sessions. Throughout this article, we examine how group coaching plays a role in facilitating a shift in one’s leader identity amid turbulent times.

As a method for engaging the transformation of a leader’s self, identity, and deeper being, this article draws from the work of Dynamic Presencing (Gunnlaugson, 2015, 2019, 2020a,b, 2021a,b, 2023) as a transformative *inner* leadership method that works with leaders in transforming their presence, presencing awareness, and consciousness—all central elements of leadership identity. By inner leadership, we mean the internal dimensions of a leader’s experience that have a significant bearing on the leader’s overall efficacy to lead well. In organizational contexts where VUCA conditions are present, inner forms of transformative leadership are essential for leading to the extent that they generate self-awareness, which develops self-knowing that drives leader efficacy (Tekleab et al., 2008).

Self-awareness provides insights into whether leader behavior is contributing to “stuckness” or movement (Higgs and Rowland, 2010). In the context of this research, self-awareness is of further importance because it is seen as a gateway to presencing. Our article provides further insights into the more subtle aspects of self-awareness in relation to presence and how Dynamic Presencing facilitates shifts in leading as a result of coming to experience our deeper presencing nature, which is key to navigating and making sense of the disruptions we face.

### 1.2 Theory U-based presencing

In recent years, among other developments, several peer-reviewed book anthologies (Gunnlaugson et al., 2013; Gunnlaugson and Brendel, 2019, 2020) have helped advance the emerging field of Theory U-based presencing. In the context of managing complexity and change, presencing is at the heart of Theory U (Scharmer, 2008), describing seven leadership capabilities that shift the way one sees, experiences, and enacts change. Eurich (2018) found that 85% of professionals are not self-aware, which in part accounts for the prevalence of ignorance and fear described by Scharmer (2018) as the absence of presencing. Absencing relates to disconnecting, denying, and distancing, which can, in certain instances, result in blaming others and the destruction of trust and relationships. In contrast, presencing is described by Scharmer (2018) as facilitating curiosity, compassion, and courage, thereby manifesting as the opening of the mind, heart, and will to deeper forms of change.

In Theory U (Scharmer, 2008), the word presencing is drawn from the words “presence” and “sensing” and relates to a “heightened sense of attention that allows individuals and groups to operate from a future space of possibility that they feel wants to emerge.” From this inner place of engagement, presencing provides a path and means for leaders to access the inner dimensions of their experience by improving their self-awareness and developing their capacity of presence, which is needed to vertically shift how and the inner place from which we perceive, know, and effectively engage our experience as leaders.

### 1.3 Dynamic Presencing

Dynamic Presencing offers an in-depth apprenticeship journey into advancing and transforming the deeper capacities, function and overall purpose of presencing practice. As a mastery approach for cultivating more advanced forms of presencing awareness that grow to become a leaderful way of being, knowing, seeing, and orienting one’s overall experience from presencing, Dynamic Presencing is focused on developing an overall sustained presencing mastery in one’s life and work.

In Dynamic Presencing, the essential nature of the leader’s self is presence. As an emerging process, leadership calls upon a leader’s ability to learn to lead from presence in the situations they are in. Learning to orient one’s experience from presence has ramifications for a leader’s identity. Here, the process of learning and discovery takes precedence over psychological identification and self-image management. Instead, presencing leadership immerses us in serving the emerging authenticating process of leading from presence. In the work of Dynamic Presencing, there is a release from the ordinary self, self-image, and its conditioned roles to establish a grounding in our deeper presencing nature—i.e., where we access the respective depths of presence in the first journey of primary presence. This shift is critical in fostering leadership development through contacting and empowering the inner dimensions of the leader.

Dynamic Presencing offers five in-depth transformational journeys to support and guide this leadership and coaching development process. These journeys include *primary presence*, *primary knowing*, *primary perceiving*, *primary communicating*, and *primary leading* (Gunnlaugson, 2020a). In this article, we will focus on the first two journeys as they provide important insight into the transformation of the leader’s ordinary self and everyday identity. Dynamic Presencing fosters a path for developing advanced levels of presencing awareness, creativity, and wisdom from within (Gunnlaugson, 2020a), addressing a void within Theory U research by helping leaders establish a sustained ontological contact with the depths of presence to develop an attunement to consciousness that is deeply embodied and practically lived in day-to-day life and work (Gunnlaugson, 2020a, 2021a, 2023).

The practice of Dynamic Presencing assists with recontextualizing conventional Western ways of knowing to learn how to sustain an embodied ontological way of knowing through a vertical shift in one’s being and presence (Gunnlaugson, 2020a). In-depth ontological engagement with our immediate experience in the presencing process is needed to overcome this Western intellectual bias. In the journey into Dynamic Presencing, a new ground state becomes active through the work of embodying and enacting presence. This shift in our state of being facilitates a generative way of seeing from our deeper presencing nature that becomes accessible to leaders (Gunnlaugson, 2020a) through the mastery of the Dynamic Presencing method. This embodied, phenomenological, and ontological approach to presencing practice reveals a new path of presencing that, in turn, sheds new insights into both Theory U as well as conventional methods of leadership development.

## 2 Training and methods

Having participated in the Dynamic Presencing leadership development training run by Olen Gunnlaugson in the Fall of 2020, six presencing practitioners situated in South Africa and the US explore our insights, voices, self-discoveries, and overall transformation through a deep collective journey. As a means of showcasing this work, this article attempts to explore some of the inner-most and essential-most impacts of Dynamic Presencing on core aspects of each practitioner’s leadership identity through different forms of presencing wisdom that emerged in our lives.

The training was conducted online over the course of 10 weeks, involving five 2-h Zoom sessions. Each training session was based on one of the five journeys, and participation required doing a preliminary reading of the respective chapter and reflective journaling, followed by group coaching of each participant’s experience in making our way through each journey. The COVID-19 pandemic created a unique opportunity for group coaching during a particularly disruptive time. As in-person meetings were not permitted during this time, we came together in the virtual space, which helped integrate our learning at the level of our leadership identity. Clutterbuck et al. (2019) argued that the benefits of group coaching include peer connection, dialogue, trust, and connection that led to an increase in self-awareness, self-confidence, stress reduction, cooperation, and wellbeing, producing a healing effect.

The Dynamic Presencing group coaching sessions were particularly instrumental in facilitating self-awareness and allowing for reflection into the multiple roles that leaders were faced with. Group coaching has been shown to contribute to facilitating wellbeing, increasing self-awareness, and allowing for a conducive environment for change and action to occur at the individual and collective levels (Nacif, 2021). This corresponds with the consensus view that group coaching has been found to be instrumental in producing a safe environment where transformative shifts can occur as a result of the input and support that others provide, along with an increase in self-awareness (Mbokota and Reid, 2022). The group coaching sessions also led to gaining new perspectives (Nacif, 2021) and reflective discoveries (Türk and Saue, 2019), which in turn helped create conditions for opening up a deeper inquiry into the merits of presencing in supporting us in facing and ultimately transforming these challenges (Aas and Vavik, 2015).

In this study, we draw on Dynamic Presencing in the leadership space through group coaching sessions. While participants were from different contexts, we nonetheless all shared the desire to develop our individual and collective leadership capabilities through the work of Dynamic Presencing. As a whole, the process and experience left a profound impact on our lives. The research team agreed to collate our insights and reflections for presentation at a peer-reviewed conference in 2021, and this work was then expanded here. The authors were all participants in the Dynamic Presencing sessions. Ethical clearance was obtained. As noted above, the first two journeys of Dynamic Presencing were chosen for the focus of the article as they illustrate the most significant data on leadership identity, which was collected qualitatively through critical reflective journaling that occurred throughout the entire Dynamic Presencing journey and beyond.

Before the commencement of the sessions, we were asked to identify and reflect on the major leadership challenges that we faced at that time. Before each session, we were asked to read the relevant chapter from the Dynamic Presencing book to become familiar with the key concepts and the overall method. In the Dynamic Presencing group coaching sessions, initial interaction included check-ins from previous journeys and instruction and explanations of the method. During each session, we were invited to engage in meditative and contemplative practices to facilitate a collective environment of presence. In these sessions, we explored the presencing method and path of presencing leadership that was introduced through each of the five journeys.

In-depth questions were posed to us during the sessions, which evoked rich reflections, further discussions, and insights for all. These explanations included thoughtful questions and time to reflect on the key aspects of the learning journey. At times, this was led through a guided experience to draw participants into a deeper level of insights and understanding. Sharing was optional, but the group members rarely chose to opt out of sharing their current experience. An environment of safety, respect and depth developed quickly among all members. Zoom breakout room sessions were critical in facilitating deeper interactions and reflections on the material that was presented. Some of the questions posed during the Dynamic Presencing sessions are listed below (see Table 1).

**Table 1:** Questions posed during the Dynamic Presencing group coaching sessions.

Describe your entry point to the journey of Dynamic Presencing.
What is your inner state of arrival as you commence the Dynamic Presencing journey?
What leadership challenges have you faced, especially as you navigated VUCA conditions in your personal and professional life?
Who are you as a leader?
How did the pandemic crisis affect the various aspects of your life as a leader?
How do you slow down, breathe, and find your center in the face of anxiety?
How do you periodically take a moment for yourself when needed at work?
How have you learned to connect to sources to address the VUCA conditions and new normal you face at work?
How do you show up when under pressure?
When are/were you most alive and internally resourced during the pandemic?
Who are you at your core / the ‘real you’—right here, right now; when you are not being influenced by the various social roles/pressures at work?
What do you need to give yourself permission to simply be?
What is blocking the real in your life at this time?
How can you be yourself as you are and draw on your innate deeper wisdom?
When do you lead well and live well?
What is taking place as you come to discover more of who you/we are?
Where do you feel most alive/what does it feel like to feel alive at work?
What does it mean to come back to you / to come home at work?
What is your quiet voice saying? Are you aware of your own trusted inner voice?
Can you distinguish, stand beside and honor your own unique voice?
Is the voice that you think is yours possibly influenced by a more conditioned voice, that you have internalized through family, social norms, and culture?
What does it mean for you to be present to your deeper unconditioned essence?
What brings you back to the source?
How can you sustain this experience?
How do you revive your felt sense of purpose and felt sense of being?
How do you connect to stillness in your life at present?
Can you imagine relating and communicating with others from this place of deep presence?
What do you need to let go of to get closer to that place?
Where does your life need you / not need you at this time?
How can your leadership be evoked from the inner place of presence moment-to-moment?
How can you learn to bring attention back to your presence-awareness in the moment?
How can you connect with presencing-awareness amid action?

The questions were usually unpacked at an individual level through quiet deliberations and then in Zoom breakout rooms with two or three participants to share and learn. These insights would then be shared in the plenary when all participants returned. Participants would have an opportunity to interact with different members and receive coaching from Olen in the group. The DP group coaching process played a role in facilitating the development and transformation of our leader identity with a noted increase in the quality of internal as well as interpersonal forms of self-, presence-, and presencing-awareness.

We were given homework at the end of the session, which would involve reflecting on the session and what came to mind/was arising. The sessions concluded with possibilities to incorporate the current journey into present life. Although all members remained at different stages and depths of the journeys, the growth as a whole was a unique shared experience. We were also invited to present a more in-depth reflection on the whole Dynamic Presencing journey when the sessions came to an end. Some of the questions posed after the Dynamic Presencing sessions are listed above (see Table 2).

**Table 2:** Questions posed post the Dynamic Presencing group coaching sessions.

How did the Dynamic Presencing journey impact you as a whole?
How did it help you in relation to orienting and navigating VUCA conditions at home or at work? How did it help anchor you?
How can you describe the emergence of the presencing self as a dimension of your presence and deeper nature?
How did you experience the journeys? What if anything shifted in your awareness, experience, or perspective from when you began?
Did you arrive at a deeper, more refined sense of who you are as a person and as a leader?
How did the work develop or deepen your practice of presencing?
What are your current presencing leadership practices and how did the training improve them?
Did you notice a different way and quality of being a leader begin to emerge for you?
How did the Dynamic Presencing journey impact you?
Were there changes?
What was the nature of the change/depth?
How did it translate into the various dimensions of your life?
Was it picked up by others?
What if anything “stuck”?
Which aspects of Dynamic Presencing appealed to you at an individual level?

Again, for the purpose of this article, the analysis of this work was broadly thematic, consolidating the experiences in accordance with the first two journeys of Dynamic Presencing presented above.

## 3 Results and discussion

### 3.1 General reflections on the Dynamic Presencing journey as a whole

Overall, the Dynamic Presencing leadership development training brought forth a deeper sense of what is possible as a leader, which served the role of grounding and empowering each participant. This occurred through gaining a new focus and gaining a significant sense of self-awareness.

*It got me to slow down in a number of ways, but also become more focused and attuned. The Dynamic Presencing journey facilitated deep inquiry into myself and the broader world. I began to slowly gain agency, inner strength, and resilience. There was a more sharpened or heightened sense of awareness, and learning to be with what-is. It is indeed a journey, which certainly did not end with the last session.* (P2).

Overall, the Dynamic Presencing journey impacted how we approached our individual paths of leadership. Reference was made to a renewed capacity for seeing from presence and experiencing some form or degree of transformation. Both personal and professional lives were impacted by a deeper connection to self, our presencing nature, and source. The Dynamic Presencing sessions were instrumental in allowing us to gain a new perspective on our experience.

*Because of what I was exposed to, my thoughts and ideas opened up a new engaging space with forthcoming and revolutionary leadership. The journeys were highly relevant to my personal life and work environment. I was able to show up from deeper locations of being. To summarize my experience with Dynamic Presencing, the past patterns and learnings contributed more to how I led and engaged with co-workers and my team as I learned to connect to deeper levels of presence.* (P3).

Looking back, the Dynamic Presencing journey was valuable at an individual level, with increasing focus, calmness, and a sense of grounding. As leaders, we were required to lead self and others, during a challenging time, which required a calm demeanor, clarity, and inner leadership at the personal level first and foremost.

*In retrospect, for me the Dynamic Presencing leadership training came at an appropriate time, when I needed to be more clear, aware and vigilant. The practices presented me with a perspective to understanding methods and tools that will allow me to be grounded in keeping me, my family and those around me safe. Under the circumstances this could only be done to the best of my ability while being calm, supportive and aware. The practices allowed me to be focused in my professional and leadership role as I engage and practice with others.* (P4).

The Dynamic Presencing sessions point to inner, subtle shifts that need to occur for leaders to attain deeper insights into who they are. These inner dimensions of leadership may be neglected in more traditional leadership development interventions. In the group coaching, we noticed an inner shift that took place collectively, which impacted positively on family life and work.

*I attended the Dynamic Presencing Journey from September to December 2020. While working through the book and attending the sessions, there was a deep, significant and subtle shift in my experience of parenting, partnering, coaching work and teaching. The inner shift was away from being anxious, unsure of my internal “footing” in relation to intra and inter subjective to an embodiment of essence and source. The shifts within a leadership coaching conversations have been profound. When both of us are coming from being essence and source, the conversation is connected and creative as we listen closely to what is and what is arising without a charged addictive focus on the future. During the journey time, there were moments of subtle shift, and now as an apprentice, intentional rituals to assist develop my practice that open up and unbounded space. Using the model of Dynamic Presencing, I realised that being real and being witness assisted my depth of being essence.* (P5).

Furthermore, insights were shared among participants in terms of deepened personal self-awareness. This was especially critical during the pandemic when demands were placed on everyone to have a robust leadership identity. This connection to source came as a natural shift from doing to being as if coming home to oneself.

*I experienced shifts on each journey, although some more profound than others to date. As I continue the work, shifts continue to be made at* var*ying paces. Interestingly, this process has not felt like work, but more of becoming me. The practice has been deepened by awareness of both phenomenological and ontological processes and attuning to these as they arise. This transformed to something on my to-do list to connect to source (ex. meditation, mindfulness,* etc.*) to breathing it into my existence. It is not something I do, but something I am, it is my being*. (P6).

Overall, the Dynamic Presencing journey collectively fostered clarity and calmness that also transferred to enhanced leadership awareness. It allowed us to focus on our immediate experience and experience a kind of high-definition quality of presencing awareness that was not possible previously. Broadly speaking, the transformative process is related to an inner shift leading to a deeper, embodied connection to self and a simultaneous heightened awareness of self, others, and the environment. The marked inner shifts from singular actions to a more persistent state of being in the group coaching brought forth a sustained contact with presence that was not only embodied (Gunnlaugson, 2020a) but also familiar and empowering in ways we had not previously imagined was possible. The Dynamic Presencing journey thus came at the right time when we as leaders needed a deeper transformative process to bring forth new insights via new practices that could shift our level of awareness and consciousness to help us more effectively navigate the emerging disruptive conditions of our VUCA world.

As a whole, the Dynamic Presencing training was powerful in grounding us to face the challenges presented by the COVID-19 pandemic, facilitating our ability as leaders to be better equipped by developing our presencing selves from the inside out. We concur with Hunter and Chaskalson: “by learning to step out of the innate human tendency to run on automatic pilot, leaders can deliberately create new options for action that can lead us through turbulent times” (Hunter and Chaskalson, 2013, p. 39).

### 3.2 The journey of primary presence

**Figure 1:**
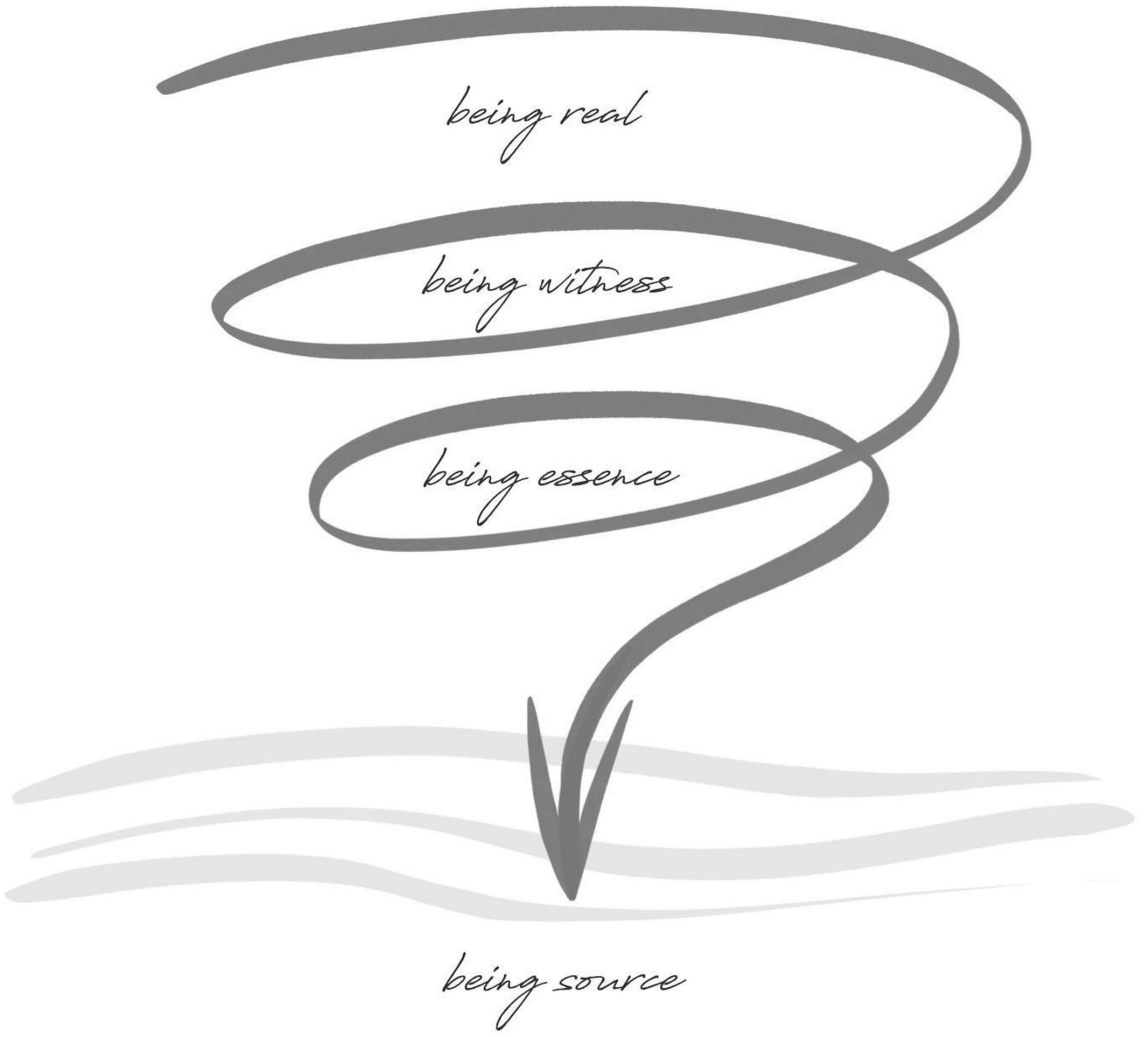
The method of primary presence in Dynamic Presencing (Gunnlaugson, 2020a).

The initial primary presence journey involves stopping the automatic pilot habits and contacting the deeper ground of presence that is drawn from and unearthed through immersion into four level depths of presence (Figure 1). This takes place with an initial surrender into being real to connect to the innate wisdom within our presencing nature. “The real then is the pervasive ground of presence that gives us cause to fully be here and arrive into the moment and presencing situation we are in” (Gunnlaugson, 2020a: p. 41). The aim is to let go of what is blocking or interfering with establishing a more existential connection with reality, such as our self-images, social identities, and societal and cultural demands on who the leader feels they should be. The intent here is to discover, then release these elements of our social conditioning so that the leader can establish deeper phenomenological contact directly with their presencing nature that lies beneath.

From here, the leader can explore witnessing their experience, opening an ontological space that is more expansive and perspectival (Gunnlaugson, 2020a, p. 45–47). Here, it becomes possible to experience a release and exhalation into a more open, distributed sense of being. “Arriving into being witness, we let go of the immediate existential intensity of our identifications but we also stay in contact with the felt textures of our experience” (Gunnlaugson, 2020a: p. 46). The felt sense of reality discovered in being witness creates conditions for the deeper inner transition to being essence. Essence is at the core of who we are, a more uniquely personal place that offers deep, sustaining nourishment from the heart of one’s life.

Traditionally recognized as the deeper seat of wisdom, essence reconnects to one’s intrinsic purpose (Gunnlaugson, 2020a, p. 49), which could also be felt as being deeply connected to one’s soul, which is core to who we are. From here, the final ontological level of source-based wisdom is accessed to guide and inform our presencing existence to fullness within (Gunnlaugson, 2020a, p. 14). In being source, we connect to the underlying formless place of creativity, stillness, and originating presence. From the descent through being real, witness, essence, and source, we move through a process of uncovering our deeper presencing nature and the different forms of presencing awareness. Overall, the four-fold journey as a whole opens up new inner vistas for our presencing self to take root inside presence directly.

As described by Skinner (2020), a well-developed leader identity is one that is internalized not only as a leader but as self, secured as and by an ontological way of being. This identity formation occurs at the individual, relational, and collective levels (Skinner, 2020). Primary presence naturally allows this leadership identity to develop through contact with our presencing self from the inside out (Gunnlaugson, 2020a).

### 3.3 Unpacking primary presence

As noted, the first journey of Dynamic Presencing began with primary presence, which relates to connecting to the four-level depths of presence through *being real, being witness, being essence,* and *being source* (Gunnlaugson, 2020a). In the group coaching, we were able to experience gradual, gentle shifts in our state of being and way of being (Gunnlaugson, 2020a) as we explored these four stages. At the depths of this journey, a connection to source deepened, despite challenges and certain forms of emotional resistance.

*During the initial time of Primary Presence, the shift was an existential one with my old thinking of connecting to source being abandoned, but to being source, sitting with who I truly am. (P6).*

The first journey in the group coaching session saw us delve into *being real.* This seemed to have a profound impact on many. As Gunnlaugson (2020a) comments, we encounter ourselves as we are without pretense, distraction, or inner division. For most of us, this exploration into the real was novel in approaching leadership development.

*The first concept taught me to be calm, aware and discerning while being strategic in my decisiveness. This concept teaches one of being real, being witness, being essence and being source. This practice I carry with me every day, particularly when I face challenges and my emotions race ahead of me, I am reminded of Primary Presence*. (P4).

It was, however, noted how challenging it was to be real, given the world that we are in. A level of self-awareness and letting go of societal pressures was an obstacle that several had to overcome on the journey.

*My entrance into the journey of presencing was not easy. A key finding in relation to primary presence was how hard it was to show up to be “real” in a world where speed and haste are necessary and rewarded.* (P1).

*What stood out was the importance of being real and how it takes courage… courage to do what exactly? Find it / speak it / claim it (back) / and return to it again.* (P2).

*For me, when I ask myself what does it mean to be real, I ask, what is my truth? When I am faced with situations that require me to be honest with who I am, without impacting my integrity, beliefs and moral standing. I approach situations with an honest and truthful disposition. The practice has taught me to not compromise who I am no matter the circumstance or situation.* (P4).

The initial journey to our “real self” was difficult and painful initially for some as it involved getting in touch with aspects of ourselves that we denied or resisted. We were able to realize that we were, in essence, often not being deeply true to ourselves. With time, through group coaching, it became easier to discover and embrace the real as a kind of ally from within rather than a threat or risk to our inner experience. Nonetheless, emotions came to the fore, which resulted in members facing the truth of their situations and lives. This opened a quality of emotional vulnerability that is described in the first journey. *Being real* is a critical first step as we find the courage to begin to come out of hiding from the subtle or less obvious ways we may be avoiding ourselves and reality (Gunnlaugson, 2020a).

*Once again, this does present challenges as I have to deal with emotions like sadness, disappointment and fear that may cause inner disruptions in the course of a day. These emotions take up time and energy which once again pose a challenge to carefree living*. (P1).

In our group coaching process, there was an effort to accept and be with reality, as it is, without the comfort of social and personal conditioning filters. This encounter with the real is a necessary step to begin the deeper apprenticeship into this form of immediate presence so that the participants can begin the journey of discovering who they are. In exploring being real, we explored a new way of seeing and relating to reality as it is—a key leadership ability.

Building from the first step of *being real*, the next step is exploring the lifeworld of *being witness*. In this awareness, we explored witnessing our thoughts, sensations, and the state of our inner body from the seat of *being witness*. The group coaching made it possible to be full of each of these experiences while witnessing them from expansive presence (Gunnlaugson, 2020a).

*Being Witness is where I intentionally allow myself to look at situations and circumstances with an objective view and mindset. I also use the term “birds eye view,” when I find myself facing situations that need me to clear my thoughts and release negative emotions that would otherwise block me from a proper and clear outcome. I review the situation and circumstance from all possible angles to allow objectivity and clarity.* (P4).

This new form of inner positionality uncovered and brought a welcome perspective-taking and meta-awareness of our experience.

*The need to confront the social identities imposed on us also stood out. We however got to see the importance of not getting trapped in this stage, by moving to the witness… the expansive presence. The session also brought up the difference between coping and thriving, which I found quite relevant given the pandemic.* (P2).

Gunnlaugson (2020a) outlines that this state of being a witness helps release us from getting caught in the dramas of our day-to-day leadership roles and responsibilities, which tends to put a strong draw down on our attention and vital energy. This is particularly critical for leaders, who might succumb to strategies of being ego-driven or preoccupied with maintaining and feeding their self-image at work.

In group coaching, witnessing awareness allowed us to step outside of our normal sense of self to see our situation and context from an expanded wisdom view of expansive presence, in turn providing an enriched perspective.

*Although the benefits of primary presence are that we connect to our true selves and learn to show up from this place in our lives, it was sometimes painful. I noticed that I would disconnect from the real me in the interest of productivity-getting my children dressed, fed, transported to and from school, delivering what is required of me at work, fulfilling my role as wife, community member and… in a demanding world I often felt that I was caught up in a vortex of giving, delivering for everyone at the cost of my personal connectedness. This convenient half-truth kept me safe from the pain of reflecting on things I did not want to confront-how should I be raising my children? Am I engaged in the right work? Am I overworked? Why should I be helping others who are capable of supporting themselves? Important questions that are relevant but expire moment to moment into the chasm of fakeness and peace that I developed as a survival strategy.* (P1).

Here, in the group coaching, some of us found ourselves reflecting on multiple, sometimes conflicting, roles and associated societal norms and pressures. Accessing the witness was also challenging despite its value:

*Holding the position of witness… does result in a certain detachment which is notably hard for my children because in order to be the witness, I am required to suspend a certain level of involvement. That said, there are undoubted benefits to living a life grounded in presencing. I am undoubted more peaceful, more connected and grounded than ever before.* (P1).

From *being witness*, we explored letting go into essence, where we started to experience our deeper, essential nature and core presence. *Being essence* was a tangible way to connect to our authentic nature and core presence, to shift to a way of working with our complex and “wicked problems” from the space of being true to ourselves, our deeper presence and way of being. In the group coaching process, this helped establish a collective sense of the inner resource of core presence to meet the growing pressure and urgency for change that pervades our times. One participant indicated that:

*It is in being essence that I come to the self-realization of putting my best foot forward. To the best of my knowledge, I endeavour to face circumstances and situations with a true inner knowledge and objective approach of being true to myself*. (P4).

The final movement led to exploring *being source*, where we contacted the very underlying foundation of being that supports who we are (Gunnlaugson, 2020a) and experienced a different way of being through collectively encountering the very ground level of originating presence.

*It is in being source that I find myself being comfortable, grounded and acknowledging my outcome be it positive or negative. It is here that I know that the outcome will bring inner achievement and growth and it also supports me in letting go. Being source allows me to journey through the different aspects and situations of life and thereby allowing me to have some kind of clarity for what the future could hold*. (P4).

On the whole, the first journey of *primary presence* served as an authenticating journey for everyone to get a taste of the four-level depths of presence and to experiment with the lifeworlds as a means to accessing this presence as a ground from which to direct one’s presencing. Collectively, this first journey provided an initiatory function in bringing the group together and opening up a path for the active cultivation of presence and our deeper presencing nature for greater leadership purposes. As each ground of presence offered its own source of support, nourishment, and overall wisdom, taken as a whole singular movement, the group explored working with *primary presence* as a method for fostering transformative leadership development.

### 3.4 The journey of primary knowing

**Figure 2:**
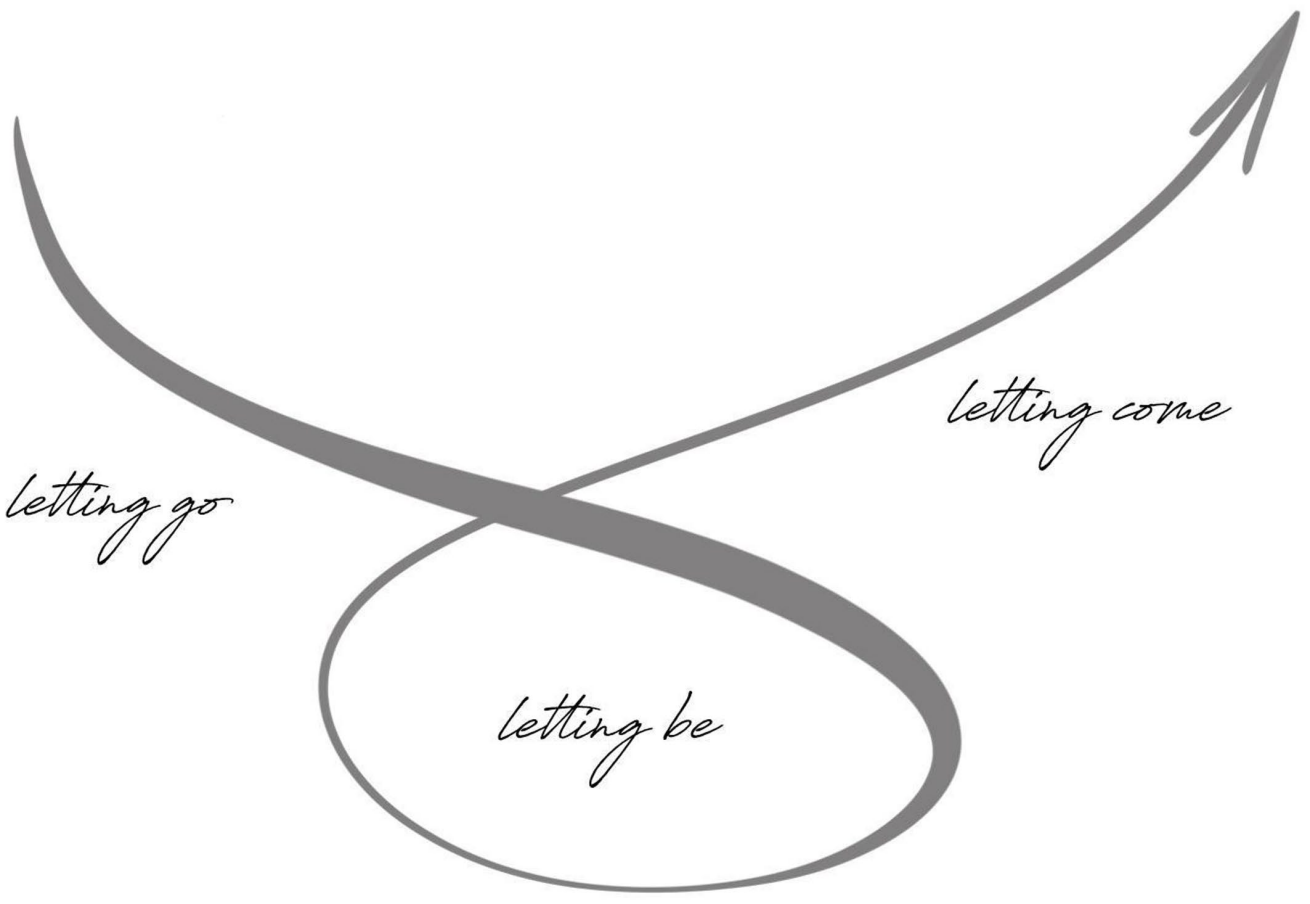
The method of primary knowing in Dynamic Presencing (Gunnlaugson, 2020a).

The second journey of primary knowing (Figure 2 below) serves as an initiating framework for leaders to learn how to stabilize both presence and presencing as a generative way of being (Gunnlaugson, 2020a). In the movement from *letting go* to *letting be* and *letting be* to *letting come*, leaders learn to slow down and ground their sense of self in the deeper ontological regions of the present moment. In this place, the learning is to be with *what-is* from a place of acceptance, receptivity, and indwelling. By slowing the urgency of action, authority is given back to presence as a means to uncovering *what-is* in a relaxed yet attentive manner.

From this presencing awareness, *letting come* allows emerging wisdom through the quality of our *letting be* (Gunnlaugson, 2020a, p. 80, 84). By embodying primary knowing, leadership takes on a dimension of remaining connected to presencing knowing from being. Shifts from *letting go* to *letting be* to *letting come* become a natural instinct within as one is inner-led from still point sourcing (Gunnlaugson, 2020a, p. 146). The identity of the leader then becomes one of active expressions, enhancing dimensional sensitivity, creative hunches, and intuition (Gunnlaugson, 2020a, p. 146).

Overall, these first two journeys of Dynamic Presencing provide an experiential way of inhabiting the rich space of presence and presencing awareness from the depths of our living experience.

### 3.5 Unpacking the journey of primary knowing

The second journey of *primary knowing* allowed us to experience a profound way of presencing knowing that is informed moment-to-moment by the quality and depth of our presence (uncovered in the first journey of *primary presence*). In the group coaching, we explored *primary knowing* as relating to the three-fold movement of *letting go* to *letting be* and finally *letting come* (Gunnlaugson, 2020a). The journey of *primary knowing* introduced a third presencing gesture, *letting be.* In Theory U (Scharmer, 2008), presencing takes place at the bottom of the U between the gestures of *letting go* and *letting come*. In this way, the group coaching *letting be* emerged as the most profound for participants, in that it was the most subtle yet also difficult to sustain.

Grounding ourselves in *being presence* became a focus to uncover this deeper *primary knowing* from the unknown regions of *letting be*. Here, our apprenticeship into presencing continued, where we explored learning to befriend the unknown as the deeper space of being that supports primary knowing from *letting be. Letting go* involved becoming aware of the busyness of our lifestyles, having to slow down and allow the head dominance to take a back seat to rediscover ourselves from a deeper place within, collectively. This was expressed as a participant indicating:

*I became more alert to the impact of the to-do-lists and* var*ious sources of stressors which had just led to a very busy state. I was also struck by the need to just be, and that it is fine to not know, and how to learn to rest in that state. The different ways of knowing, and being able to see how the head takes too much dominance also made an impact. It was quite an intense realization*. (P5).

This aligns with awareness of not giving in to the urge to be overly preoccupied with wanting to take action or focus obsessively on the future (Gunnlaugson, 2020a), something the Theory U approach to presencing sometimes fosters. From a leadership perspective, this re-balancing one’s presence in the present becomes critical, as we often find ourselves rushing to make decisions without taking a pause and allowing for the different dimensions of knowing to blossom on our own terms and timelines.

We discovered that we need to learn to refine the *letting go* as leaders and to learn to re-orient our sense of who we are away from the known, tried and tested, safe options. Exploring an immersion in what is unfolding in the moment helped participants appreciate the value of learning to lead from presence here and now and to let one’s primary knowing germinate from these depths of presence, accessible through the gesture of *letting be*. Here, a participant noted a connection with intuition:

*What was missing was the connection of my intuition, rather than it being a separate entity within my life. One of my life sayings was a Latin phrase of “Puto ergo sum,” translated “I think therefore I am.” Within the journey of Primary Knowing there was a drastic shift in hearing “I am before I think.” Learning to allow knowledge in this new way became a relief on my innate goal-oriented mindset of doing and wrapped myself in simply being, trusting the process*. (P6).

Participants experienced a paradox in learning to let go as well, to achieve new insights in ways that were unfamiliar to them. In doing so, the arising new began to emerge naturally throughout daily life.

*In a world of uncertainty and complexity, it seems contra-indicated to let things go to get ahead. But as Olen described, the point of letting be allows a deeper more trusted source of knowing to emerge. This experience of being is profound, uncertain and sometimes a little unnerving. My letting go, organically facilitated the attuning to what-is, which resulted in insights emerging for me during meditation, and walks in nature. Primary knowing started to emerge powerfully and clearly, and the discerning of the new came with excitement for me.* (P1).

*Letting go* as a surrender helped us release ourselves into *letting be* as a way of working more stably from the ground of presence moment-to-moment (Gunnlaugson, 2020a). A participant described a sense of relief emerging:

*The noise began to fall away. This did require me to cut and release my attachment to outcomes that did not come naturally for me. The process has since become more fluid and connected to my presence of letting be. This shift has been particularly impactful for me as a leader in that less and less shakes my external and internal selves, but a calmness that is open to leading others into this same space.* (P6).

Many leadership development efforts focus on developing emotional intelligence. This study adds the unique dimensions of consciousness to deepen our awareness with respect to self and others. In the group coaching, a participant described becoming aware of the deeper groundless state, which was critical to moving forward. It was highlighted that

*The idea of being at home in the groundless state, being able to suspend the intellect, and just slow down and stop hit a nerve….* (P2).

Another participant, however, indicated an initial fear:

*There was a fear that came with this shift in that “groundless ground,” but much like being the chrysalis, a comfort was present that it was the exact place I was to be, and the fear mostly dissipated. This journey in particular led me to personally focusing on both light and shadow work at a soul level.* (P6).

An appreciation for stillness emerged in the group coaching, given the VUCA conditions, which had almost become a norm for us during this time. A participant shared that the idea of being able to connect to source and listen from where things are still and quiet offered a new place to learn to lead from in *primary knowing*, participants began to taste the possibilities of presencing as a way of being, which slows us down into the depths of presence to learn how to anchor our awareness from these depths. This is a critical insight for leadership, as we may not be aware of how impactful slowing down and embracing stillness can be for developing wisdom and being in a resourceful state to make better quality decisions.

This aligns with the experience of slowing ourselves down and breathing into the face of anxiety, fear, or whatever deeper emotions may arise, as we explored in the group coaching. Reflections centered on how to put your being first and learning to rest and trust in this process that has your back amid action and reflection. Participants were struck by questions in the coaching sessions relating to deep nourishing rest through sustained contact with deeper forms of presence and our presencing nature, particularly how that felt in regenerating their lives in ways that were impactful. As leaders, we sometimes forget to value, let alone understand, how to connect with rest as a practice foundation for self- and presence-renewal, as well as more sustained generativity from within. Another participant was deeply impacted by the effortless nature of primary knowing and how we are under so much pressure to perform as leaders. How do we reconcile this? In the group coaching, this was brought about by reflections on the need to schedule dormant periods in one’s work week to practice *being presence*—the aim of the first journey of primary presence. This also contributed to work–life balance deliberations and helped everyone appreciate leadership in renewed ways through this presencing exploration.

Overall, the primary knowing phase created a deeply regenerative opportunity for self-awareness to develop directly from the ontological depths of presence and being—a new experience for all participants. In the group coaching, participants described gaining new knowledge and insights into the depth dimensions of their presencing selves. These discoveries began as ideas but soon became experiential with practice. There was a gradual sense of growing and developing our presence and presencing nature through the challenging experiences, situations, and circumstances that were in our daily lives at this time. Curiously, the training developed a collective resolve and inner strength, a feeling of being able to face the deeper pandemic challenges by learning a new presencing way of being that in the beginning was unfamiliar, yet through the course of the group coaching, grew uniquely for each participant in ways that empowered our voices and wisdom as emerging leaders.

## 4 Concluding remarks

Together, through group coaching and individually, we sought to explore the unfolding of transformative leadership development through our collective journey into Dynamic Presencing. As a sanctuary from the disruptive times of our global pandemic, our Dynamic Presencing training (Gunnlaugson, 2020a) facilitated significant gradual, though no less vertical, shifts in our overall self and presencing awareness, fostering a greater appreciation for the cultivation of presencing mastery in our lives and work as leaders. As a whole, Dynamic Presencing served as a critical enabler in anchoring us in different wisdom grounds of presence. By learning to embody a deeper understanding of leadership efficacy and growing our confidence and resourcefulness for facing the VUCA conditions of our global world, our inner leadership journey became a trusted path of transformative leadership development. Our reflections in this article highlight how cultivating new forms of presencing awareness through internal forms of self-awareness, confidence, and resourcefulness central to the leader experience facilitated core capacities and conditions for developing an inner foundation to support and develop our overall presencing leadership, a path that everyone continues to explore in their work and lives to this day.

Following this training and our reflections on it improved self-awareness for some participants, improved their decision making with an increased awareness of the inner place they are taken from, and increased clarity of presenced seeing into the depths of the alternatives that are available at the moment. In a related way, increased self-insight, self-understanding, and self-wisdom developed for other participants through a deepened self-awareness of their intrinsic values, which in turn helps in sustaining creativity at the level of their being. A more accepting attitude toward change (both in pace and scope), deepened internal locus of control, and greater tolerance of ambiguity in one’s life and work, followed by the overall shift in our center of gravity toward a stronger identification with our presencing nature, which is inherently process-based, not identity-based, are therefore far more effective at working with and through change. These developments were reported as particularly significant in helping us more effectively navigate VUCA conditions in our life and work, as our deeper presencing nature is at home in the volatility, uncertainty, complexity, and ambiguity of situations. Related to this was a greater efficacy and resilience in managing stress, an increasing issue for leaders/managers, particularly during the pandemic and in our post-pandemic world.

Drawing from these and related impacts of the Dynamic Presencing leadership development training, in carving out our own unique sense of presenced leader identity through the training and group coaching, in retrospect, this aspect was critical in helping us find a meaningful way through a particularly disruptive time in our lives. Overall, the Dynamic Presencing training and group coaching facilitated a significant collective shift in our collective way of thinking, feelings, and actions at the deeper levels of our being, presence, and humanity, something that continues to serve as a deep inner reference point to lead us in our day-to-day lives.

## Data availability statement

The data (anonymized) supporting the conclusions of this article can be made available by the authors, upon request.

## Ethics statement

The studies involving humans were approved by UKZN Humanities and Social Sciences Research Ethics Committee. The studies were conducted in accordance with the local legislation and institutional requirements. The participants provided their written informed consent to participate in this study.

## Author contributions

CP: Writing – original draft, Writing – review & editing. PD: Writing – review & editing, Writing – original draft. JS: Writing – original draft, Writing – review & editing. CrL: Writing – original draft, Writing – review & editing. NM: Writing – review & editing, Writing – original draft. ChL: Writing – original draft, Writing – review & editing. OG: Writing – review & editing, Writing – original draft.
